# Secondary CNS Lymphoma in an Immunocompromised Patient: A Diagnostic Dilemma

**DOI:** 10.7759/cureus.69468

**Published:** 2024-09-15

**Authors:** Muhammad Qasim Naeem, Muhammad Atif Naveed, Ambar Ambreen, Abdullah Imran, Muhammad Asad Ullah

**Affiliations:** 1 Radiology, Shaukat Khanum Memorial Cancer Hospital and Research Centre, Lahore, PAK; 2 Diagnostic Radiology, Dr. Ziauddin Hospital, Karachi, PAK; 3 Radiology, Shaukat Khanum Memorial Cancer Hospital and Research Centre, Karachi, PAK

**Keywords:** cerebral abscess, cns lymphoma, diffuse large b cell lymphoma (dlbcl), hiv/aids, immunocompromised

## Abstract

Primary lymphoma can occur in the brain and is restricted to the central nervous system (CNS). Secondary lymphoma involves the CNS after affecting other organs in the body. The prognosis is worse for secondary CNS lymphoma. Early diagnosis and treatment are mandatory. We report a case of secondary CNS lymphoma that was misdiagnosed as a cerebral abscess because of its unusual features.

## Introduction

Primary lymphoma can occur in immunocompetent and immunocompromised patients. It is strictly restricted to the brain, spinal cord, or meninges [1]. In contrast, secondary CNS lymphoma occurs as a systemic manifestation of a primary lymphoma elsewhere. This is also known as metastatic lymphoma [2]. Secondary CNS lymphoma is more common in untreated AIDS or Burkitt lymphoma cases as demonstrated by flow cytometry analysis of cerebrospinal fluid (CSF) [3]. CNS involvement frequently emerges early in the treatment of patients with aggressive NHL, implying that subclinical involvement of the CSF may be present at diagnosis [4]. The risk of secondary CNS lymphoma depends on the histologic subtypes; for non-Hodgkin lymphoma (NHL), it varies between 2% and 27% [5,6], while those with Hodgkin lymphoma have an exceptionally low risk of <1% [5]. Lymphoma can spread to the CNS through hematogenous dissemination, contiguous structural invasion, or extension along the neural pathways [7]. Parenchymal involvement is more common than meningeal involvement; however, simultaneous parenchymal and meningeal involvement is not uncommon in secondary CNS lymphoma [8]. Due to its variable imaging appearances, the diagnosis remains challenging [2,7,9]. Thus, a reporting radiologist must be more cautious and aware of atypical presentations. We present an unusual case of biopsy-proven secondary CNS lymphoma, which was initially reported as an abscess due to its atypical presentation.

## Case presentation

A 35-year-old man began experiencing weight loss a few months before presenting to the clinic in April 2023. He developed a dry cough, which improved with oral antibiotics. After three months, he started experiencing fever, dyspepsia, and vomiting. A chest radiograph revealed a cavitating lesion in his lungs, and he was started on antituberculosis (ATT) medications. Two months later, he developed abdominal pain and vomiting and was diagnosed with an acute abdomen. His baseline investigations revealed lymphocytopenia and raised inflammatory markers (Table 1).

**Table 1:** Baseline investigations. Note lymphocytopenia and raised inflammatory markers. MCV: mean corpuscular volume; HCT: hematocrit; ALT: alanine transaminase; AST: aspartate aminotransferase; GGT: gamma-glutamyl transferase; CRP: c-reactive protein; ESR: erythrocyte sedimentation rate; LDH: lactate dehydrogenase

Complete blood count			
Parameters	Results	Units	Reference Range
Hemoglobin	12.1	g/dL	13.2–16.7
MCV	80.4	fL	77.8–96.2
HCT	37.8	%	40.1–49
Platelets	212	x10^3/µL	150–450
WBC	7.34	x10^3/ µL	4.52–10.93
Neutrophils	85.9	%	40.7–70.7
Lymphocyte	2.9	%	20.2–40
Monocyte	9.7	%	3–10
Eosinophil	1.1	%	0.5–6
Basophil	0.4	%	0.2–1.1
Liver Function Tests			
ALT	58	U/L	<45
AST	44	U/L	<35
GGT	53	U/L	12–62
Alkaline Phosphatase	107	U/L	50–116
Total Bilirubin	0.28	mg/dL	0.3–1.0
Albumin	3.4	g/dL	3.5–5.2
Globulin	4.17	g/dL	2.3–3.4
Serum Inflammatory Markers			
CRP	57	mg/dL	<5
ESR	19	mm/1^st^ hr	0–15
Ferritin	2,071	ng/mL	20
LDH	355	U/L	135–225

He underwent laparotomy, during which surgical findings revealed intestinal perforation, necessitating bowel resection and anastomosis. Histopathology revealed diffuse large B-cell lymphoma (DLBCL). Fluorescence in situ hybridization (FISH) did not detect CMYC or idiopathic guttate hypomelanosis/B-cell leukemia/lymphoma 2 (IGH/BCL2) gene rearrangements. He also tested positive for HIV and CMV during the same admission (Table 2).

**Table 2:** Viral markers obtained at baseline. PCR: polymerase chain reaction; CMV: cytomegalovirus; HBsAg: hepatitis B surface antigen; HCV: hepatitis C virus. HIV was found to be reactive and PCR of HIV and CMV were obtained during the same admission.

Parameters	Results	Units
HIV Viral Load (PCR)	62,000	copies/mL
CMV Viral Load (PCR)	51,40,000	copies/mL
HBsAg	Non-reactive	
Anti-HCV	Non-reactive	

A bone marrow biopsy showed normal trilineage cells. A peripheral smear was obtained which was abnormal (Table 3).

**Table 3:** Peripheral blood smear.

RBC Morphology	Anisocytosis, Elliptocytes, Target Cells, Basophilic Stippling, Polychromasia
Differential Leukocytes	Neutrophils 91%, Lymphocytes 03%, Monocytes 06%
Nucleated RBCs	19/100 WBCs
WBC Morphology	Increased with neutrophilia and left shift
Circulating Blasts	None
Atypical Cells	None

T-helper and T-suppressor cell counts were also low (Table 4).

**Table 4:** Differential counts of T-lymphocytes.

CD4/CD8 Count			
Parameters	Results	Units	Reference Range
WBC	6,770	--	--
Total Lymphocytes Count	100	--	--
Average T-Lymphocytes CD3+	26	/µL	668–2,291
T-Helper Lymphocytes (CD3+CD4+)	2	/µL	433–1,692
T-Suppressor Lymphocytes (CD3+CD8+)	11	/µL	147–1,068
(CD3+CD4+/CD3+) Ratio	7.6	%	28–64

Following surgery, he underwent a repeat endoscopy and colonic biopsy because of ongoing abdominal pain, but biopsies showed normal bowel mucosa. He was started on R-CHOP (cyclophosphamide, doxorubicin, prednisone, rituximab, and vincristine) therapy. A few days later, he experienced loss of consciousness and fever. CSF analysis obtained was also abnormal (Table 5).

**Table 5:** CSF analysis.

Parameters	Results	Units	Normal reference range
Albumin	29	mg/dL	11–35
Proteins	91.7	mg/dL	15–45
Glucose	35	mg/dL	40–70
LDH	49	mg/dL	0–40

**Figure 1:**
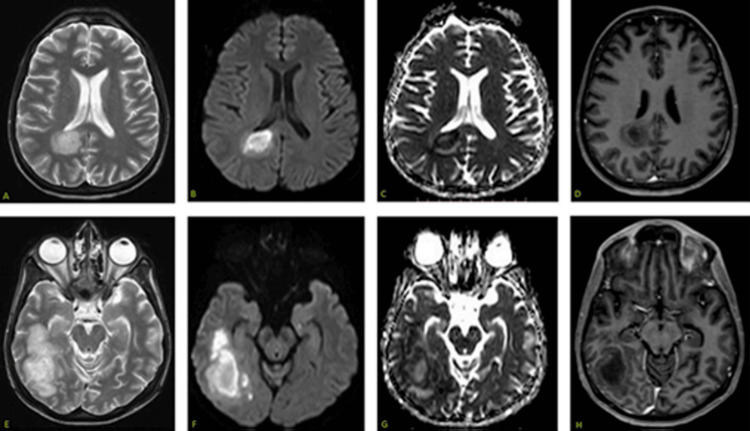
An MRI of the brain with IV contrast shows T2 hyperintense lesions with diffusion restriction and very weak peripheral post-contrast enhancement in the splenium of the corpus callosum extending to right periventricular white matter (A-D), and right temporal lobe (E-H).

An MRI showed two lesions in the brain: one in the splenium of the corpus callosum extending into the periventricular white matter adjacent to the occipital horn of the right lateral ventricle and a second lesion in the right temporal lobe, reported as cerebral abscesses (Figure 1).

**Figure 2:**
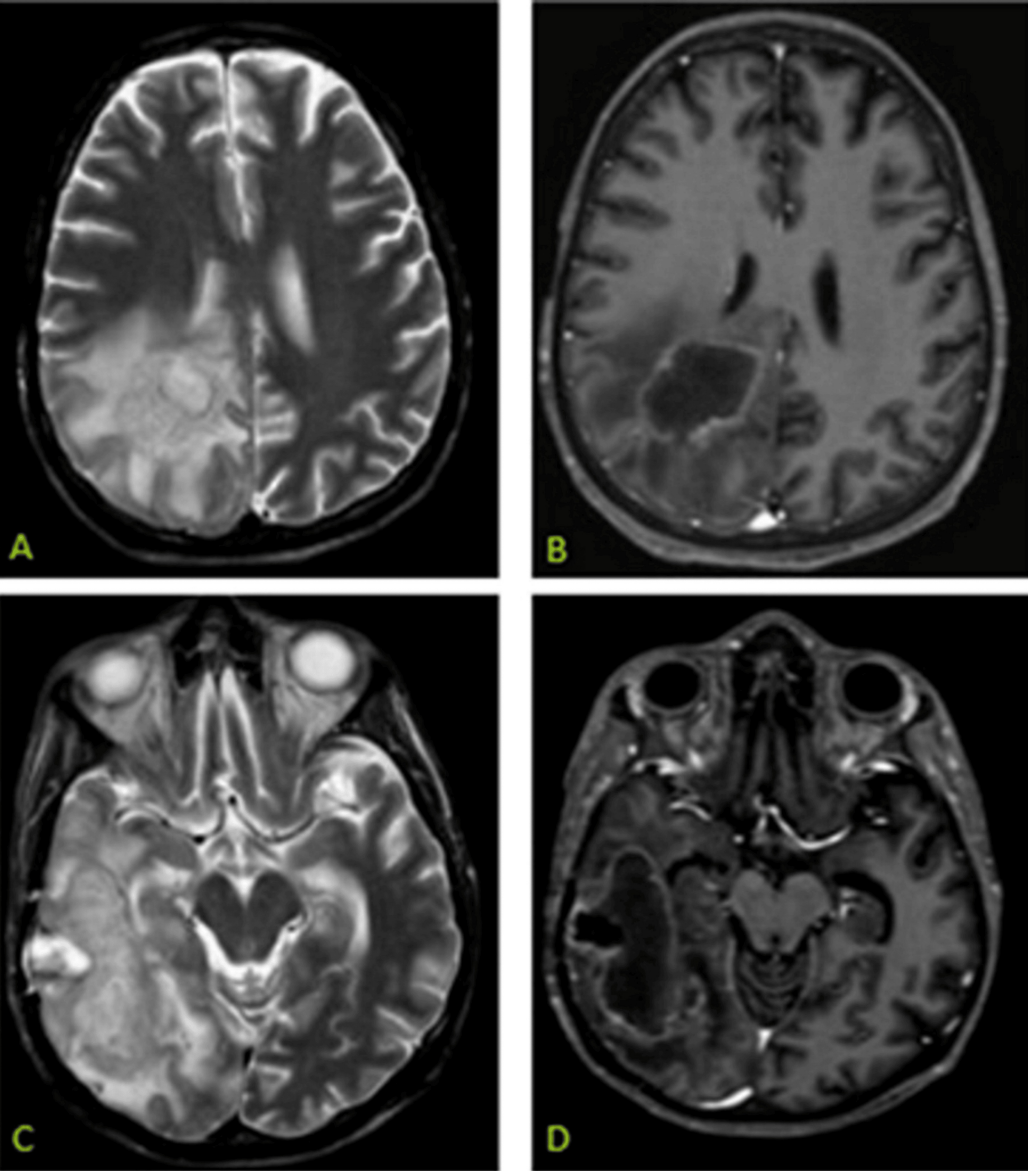
An MRI scan obtained after one month. T2 and post-contrast images show an increase in the size of the lesions and corresponding vasogenic edema.

Despite antimicrobial, antiviral, and antifungal treatment, his condition worsened, requiring mechanical ventilation. A repeat MRI one week later showed mild progression of the lesions. The neurosurgical team was consulted for a biopsy of the lesions. The intracranial biopsy revealed a high-grade neoplasm containing large lymphoid cells with high mitotic activity. CD20 and PAX5 were positive, and the Ki67 index was 70%. During the hospital admission, he developed a sacral bedsore that cultured positive for multidrug-resistant Klebsiella pneumoniae. A month later, a follow-up MRI showed further progression (Figure 2). Unfortunately, despite efforts, his condition continued to deteriorate, and he succumbed to death.

## Discussion

The lymphomatous involvement of the CNS in immunocompromised patients has been a challenging diagnosis in imaging. Different patterns of signal abnormalities have been noted and described in earlier studies, including ring enhancement and complete solid enhancement [2,9-13]. Lymphoma can involve any area of the brain, and various clinical presentations can be observed depending on the area involved. Leptomeningeal disease is frequently encountered in secondary CNS lymphoma [13]. Because of its variable presentations, a CT examination alone is insufficient; an MRI with contrast enhancement is always required to evaluate the extent of the disease fully.

Notably, MRI imaging cannot differentiate between primary and secondary CNS lymphoma, as both have similar imaging features. Therefore, if CNS lymphoma is suspected on imaging, it should be followed by a whole-body F-18 fludeoxyglucose (FDG) PET scan to evaluate the extra-CNS extent of the disease. The risk of CNS relapse also depends on the histologic grade, advanced disease, multiple extranodal sites, and the primary site of origin. In one study, parenchymal disease was less commonly encountered in secondary CNS lymphoma, accounting for one-third of the cases, while leptomeningeal disease was much more common [7,12]. However, the study by Malikova et al. demonstrated an increased incidence of parenchymal disease compared to leptomeningeal disease [2]. Supratentorial cerebral parenchyma was much more frequently affected, and, in most cases, there was diffuse enhancement and complete diffusion restriction [2]. Secondary CNS lymphoma has a poor prognosis, with a median survival rate of four to five months [2].

In our patient, a unique imaging appearance was observed that has not been reported or is very rarely observed after initiation of treatment [2]. A baseline MRI of the brain showed two lesions: the first lesion in the splenium of the corpus callosum and adjacent periventricular white matter, and the second lesion in the right temporal lobe. These lesions demonstrated a low signal on T1-weighted images, a high signal on T2-weighted images, and fluid-attenuated inversion recovery (FLAIR) sequences. There was intense diffusion restriction with internal heterogeneity, which was somewhat unusual, along with a very thin rim of peripheral enhancement. The case was initially misdiagnosed as a cerebral abscess. As the patient’s condition progressively declined, the neurosurgical team was contacted for a cerebral biopsy. The biopsy results were consistent with DLBCL. A repeat MRI after a month showed the progression of the disease. A few days later, the patient succumbed to his illness.

## Conclusions

In summary, patients with previously known systemic lymphoma who experience a declining neurological status should prompt physicians to perform early neuroimaging. Because of its variable presentation, neuroimaging may mimic other solid or peripherally enhancing lesions, and radiologists should consider the differential diagnosis of secondary CNS lymphomatous involvement. In challenging cases, invasive testing, such as brain biopsy, remains the gold standard.
